# Insecticide resistance status of the malaria mosquitoes: *Anopheles gambiae* and *Anopheles funestus* in eastern and northern Uganda

**DOI:** 10.1186/s12936-018-2293-6

**Published:** 2018-04-06

**Authors:** Michael Okia, David F. Hoel, James Kirunda, John Bosco Rwakimari, Betty Mpeka, Denis Ambayo, Ananya Price, David W. Oguttu, Albert P. Okui, John Govere

**Affiliations:** 1 USAID Uganda Indoor Residual Spraying Project Phase II, Abt Associates Inc., Kampala, Uganda; 2 Lee County Mosquito and Hyacinth Control District, Lehigh Acres, FL USA; 3 USAID Zimbabwe Assistance Program in Malaria, Harare, Zimbabwe; 4 Vector Control Division of the Ministry of Health, Kampala, Uganda; 5 National Malaria Control Programme, Ministry of Health, Kampala, Uganda; 6 University of Witwatersrand, Johannesburg, South Africa; 7 Uniformed Services University of the Health Sciences, Bethesda, MD USA

**Keywords:** *Anopheles gambiae*, *Anopheles funestus*, Uganda, Insecticide resistance, Oxidase, Intensity bioassay

## Abstract

**Background:**

Uganda’s malaria burden includes the sixth highest number of annual deaths in Africa (10,500) with approximately 16 million cases (2013) and the entire population at risk. The President’s Malaria Initiative has been supporting the malaria control interventions of indoor residual spraying (IRS) and distribution of long-lasting insecticidal nets (LLIN) in Uganda since 2007. These interventions are threatened by emerging and spreading insecticide resistance, known to exist in Ugandan malaria vectors. Pyrethroid insecticides have been used in agriculture since the early 1990s and in IRS programmes from the mid-2000s until 2010. A universal LLIN coverage campaign was executed in 2013–2014, distributing pyrethroid-treated LLINs throughout the country. This study investigated insecticide susceptibility, intensity, and oxidase detoxification in *Anopheles gambiae* sensu lato and *Anopheles funestus* to permethrin and deltamethrin in four eastern Ugandan sites.

**Methods:**

The susceptibility status of *An. gambiae* and *An. funestus* to bendiocarb, permethrin and deltamethrin was determined using the CDC (Centers for Disease Control and Prevention) bottle bioassay. Presence of oxidative enzyme detoxification mechanisms were determined by pre-exposing mosquitoes to piperonyl butoxide followed with exposure to discriminating doses of deltamethrin- and permethrin-coated CDC bottles. Resistance intensity was investigated using serial dosages of 1×, 2×, 5× and 10× the diagnostic dose and scored at 30 min to determine the magnitude of resistance to both of these LLIN pyrethroids. Testing occurred in the Northern and Eastern Regions of Uganda.

**Results:**

*Anopheles gambiae* and *An. funestus* were fully susceptible to bendiocarb where tested. *Anopheles gambiae* resistance to deltamethrin and permethrin was observed in all four study sites. *Anopheles funestus* was resistant to deltamethrin and permethrin in Soroti. Oxidative resistance mechanisms were found in *An. gambiae* conferring pyrethroid resistance in Lira and Apac. 14.3% of *An. gambiae* from Tororo survived exposure of 10× concentrations of permethrin.

**Conclusions:**

Both *An. gambiae* and *An. funestus* are resistant to pyrethroids but fully susceptible to bendiocarb at all sites. Susceptibility monitoring guided the Ministry of Health’s decision to rotate between IRS insecticide classes. Intensity bioassay results may indicate encroaching control failure of pyrethroid-treated LLINs and should inform decision-makers when choosing LLINs for the country.

## Background

Renewed interest in malaria elimination has led to the scale-up of vector control measures in sub-Saharan Africa (SSA) [1]. Given that IRS and LLINs, the most effective malaria prevention methods, rely heavily on insecticide use, it is critical to monitor vector resistance to insecticides [2]. Both LLINs and IRS are the priority malaria prevention interventions in Uganda. Uganda attempted a universal LLIN coverage campaign (one LLIN per two people) in 2014 after distributing over 22 million LLINs provided by the Global Fund to Fight AIDS, Tuberculosis and Malaria (GFATM), USAID/PMI, World Vision and other partners, distributing LLINs over most of the country. With support from USAID/PMI and the Department for International Development (DFID), IRS was implemented in 10 districts in northern Uganda from 2007 to 2014 and is currently performed in 14 new highly malaria-endemic districts in northern and eastern Uganda. All these expanded vector control measures coupled with use of pesticides in agriculture exert insecticidal pressure on local malaria vector mosquitoes, which may accelerate the development and spread of insecticide resistance. Past studies conducted in Uganda have confirmed that *Anopheles gambiae* sensu lato (hereafter *An. gambiae*) was susceptible to carbamate and organophosphate insecticides, however, pyrethroid resistance was detected in Apac, Lira, Soroti and Tororo District surveillance sites, although there was wide variation in susceptibility to the different pyrethroids. Pyrethroid and dichlorodiphenyltrichloroethane (DDT) resistance in both *An. gambiae* and *Anopheles funestus* is a growing problem in the country [3–9] and has become a major malaria control concern and a threat to the success of insecticide-based malaria vector control programmes, not only in Uganda, but in most of sub-Saharan Africa (SSA) [10–12]. Insecticide resistance will definitely affect the achievement of the goal of Uganda National Malaria Control Programme (NMCP) for “A Malaria Free Uganda” [13, 14]. Thus, insecticide resistance management is presently one of the main focus areas of the WHO Global Malaria Programme [15].

In Uganda, the level of *An. gambiae* vector resistance against bendiocarb, deltamethrin and permethrin was investigated in four sites (Apac, Lira, Soroti and Tororo Districts) that are located in highly malaria-endemic rural areas of northern and eastern Uganda [16]. These four sites are situated in and next to the present-day IRS operational zone. The CDC bottle bioassay [17, 18] was used for determining insecticide resistance status, mechanisms and intensities in the major malaria vectors *An. gambiae* and *An. funestus* to three public health insecticides used for IRS, two of which are used in the impregnation of LLINs. Two of the current surveillance sites, Apac and Tororo, were also a part of the national malaria vector resistance studies conducted by the MoH and Malaria Consortium using WHO tube bioassays [19, 20] in 2009 and by MoH and Abt Associates in 2011, 2013 [3] and 2015.

This paper presents the findings of recent studies on the insecticide susceptibility status of *An. gambiae.* and *An. funestus* against bendiocarb, deltamethrin and permethrin in Apac, Lira, Soroti and Tororo.

## Methods

### Study sites

**Fig. 1:**
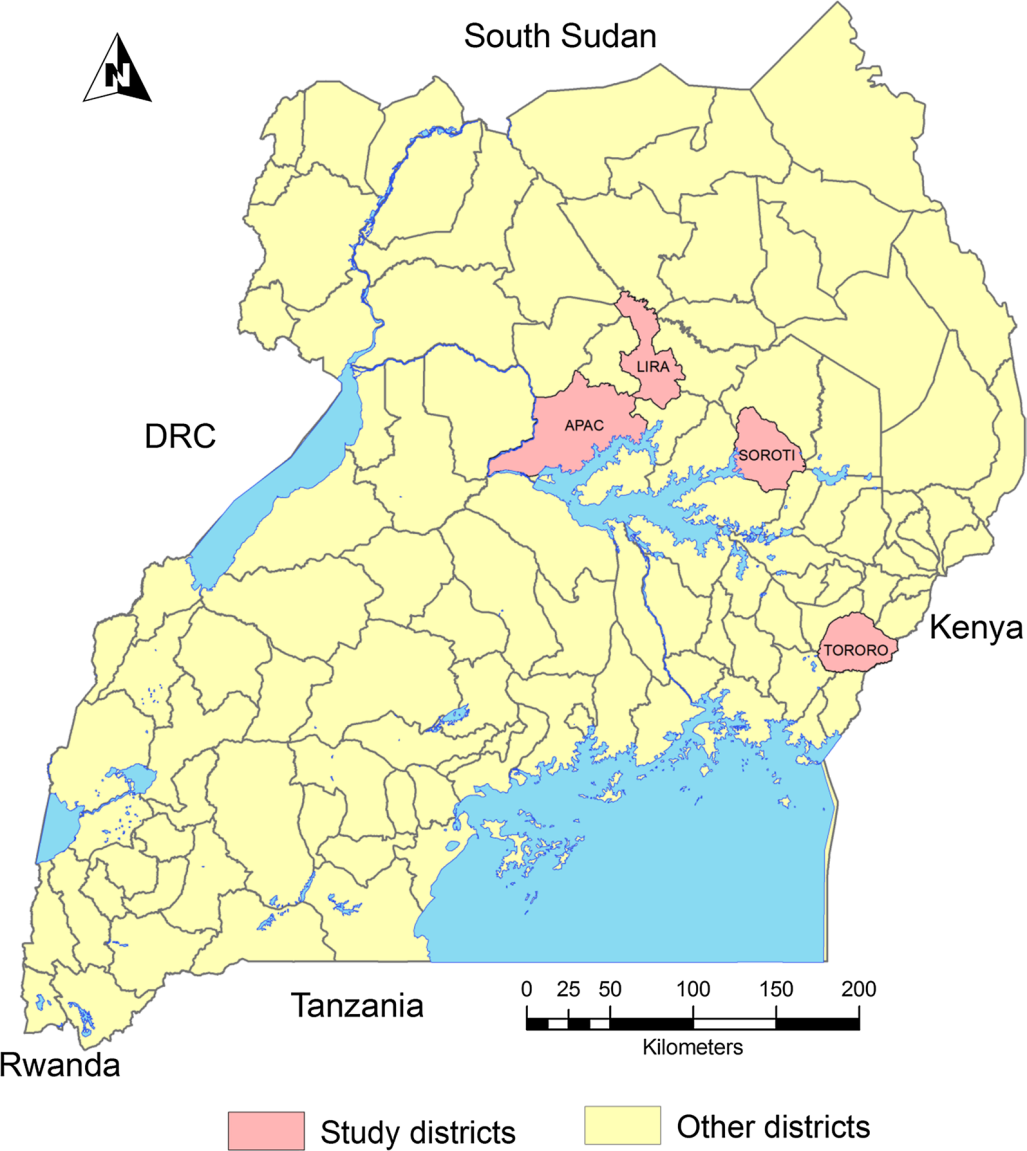
Map of Uganda showing susceptibility assay study districts

The study was conducted in Lira and Tororo (current IRS districts), Apac (former IRS district) and Soroti (a non-IRS district), all rural districts (Fig. 1). Apac, Lira, and Soroti Districts are north and northeast of Lake Kyoga in northern Uganda and Tororo District is in southeast Uganda along the Kenyan border (Fig. 1). Apac, Lira, and Soroti Districts consist of mostly flat country with small, scattered rolling hills with rock and boulder outcroppings. Savannah woodland with swamps and wetlands are common in these three districts with villages dispersed throughout. Tororo District is hilly over much of the countryside and Tororo town has a volcanic core within city limits. Tororo District large boulders scattered throughout; the Kenyan highlands are to the east and visible from town. Riverine zones and lowlands in Tororo District are planted in rice.

Apac District is located approximately 262 km north of Kampala, the capital city of Uganda. Apac lies between longitudes 32° E and 34° E and latitudes 2° N and 3° N, at an average altitude of 1150 m above sea level with 9% of the district consisting of open swamps. Lira District is located approximately 364 km north of Kampala and lies between latitudes 1° 21′N and 2° 42′N and longitudes 32° 51′E, 34° 15′E, at an average altitude of 1200 m above sea level. Soroti District is located 347 km northeast of Kampala and lies between longitudes 30° 01′E and 34° 18′E and latitudes 1° 33′N and 2° 23′N, at an average altitude of over 1250 m above sea level. Tororo District is located 205 km northeast of Kampala and lies between 0° 45′N, 34° 5′E in Eastern Uganda on the Kenyan border.

All four districts have stable, perennial malaria transmission with malaria prevalence rates ranging from 37 to 63% [21]. All sites experience two malaria peaks following two rainy seasons which occur from March to May and again from August to October with intermittent rain in-between. Annual rainfall totals range from 1200 to 1800 mm. Temperatures in northern Uganda (Gulu climate data) range from an average monthly high of 25 °C in February to a low of 22.3 °C in July [22].

Study district populations according to the National Population and Housing Census (2014) are Apac, 368,000; Lira, 408,000; Soroti, 297,000; and Tororo, 517,000. All district populations are > 75% rural. Three types of house predominate in these districts: permanent houses with plastered and painted wall surfaces, semi-permanent houses with mud and wattle walls and tin roofs, and temporary houses with mud and wattle walls and thatch roofs that make up the majority of houses in all districts. Major agricultural products in all test districts include sweet potatoes, cassava, groundnuts, beans, maize, millet and sorghum, and other recently introduced crops such as rice, sunflower, soybeans, and citrus fruit. Horticultural crops serve both as food and cash crops. Livestock, mainly cattle, goats, sheep, rabbits, swine, and poultry are raised [23].

### Mosquito collections

Anopheline mosquitoes were collected from larval breeding sites or as adults from indoor resting collections in Apac, Lira, Soroti and Tororo (Fig. 1). Trained mosquito collectors used test tubes to collect indoor resting mosquitoes between 5.00 a.m. and 7.00 a.m. in June 2015 following a verbal consent from house owners. Larvae were collected from different types of breeding habitats (roadside ditches, marram/brick/sand pits, ponds, puddles, hoof prints) using dippers/scoops and reared in an insectary at 28 ± 2 °C room temperature and 75–80% relative humidity. Individual adult *Anopheles* mosquitoes collected from the field were identified using a simplified morphological key adapted from Gillies and Coetzee [24].

### Insecticide susceptibility tests

The CDC bottle bioassay [17, 18] was used for detecting resistance to insecticides. The bioassay is, in principle, the same as the WHO paper tests [19, 20]. Morphologically identified non-blood fed 2–5 day old *An. gambiae* and *An. funestus* adult mosquitoes were tested with the standard CDC bottle bioassay using diagnostic doses of 12.5 and 21.5 μg/bottle of deltamethrin and permethrin, respectively at a diagnostic time of 30 min applicable to *Anopheles* mosquito populations [25]. The study teams treated bottles using diluted insecticide prepared and brought from CDC Atlanta. Four replicates (bottles) of approximately 25 mosquitoes served to monitor susceptibility status with another 10–15 added to a fifth acetone-only treated control bottle. The diagnostic dose for each insecticide was determined as the minimal amount of an insecticide needed to kill all susceptible mosquitoes at 30 min. A 30 min exposure in treated bottles is considered the most critical value, because it represents the threshold between susceptibility and resistance. Mosquitoes were considered dead when they could no longer stand, were immobile, and slid along the curvature of the test bottle. Mortality was recorded every 15–120 min, if necessary, for treated-bottle survivors. At the end of 120 min, control bottle mortality was scored [18].

### Insecticide resistance mechanism tests

Insecticide resistance mechanisms were investigated with a synergist, piperonyl butoxide (PBO), in *An. gambiae* and *An. funestus* to two insecticides commonly used in Uganda malaria control programme interventions: deltamethrin (IRS and LLINs) and permethrin (LLINs) [17, 18]. Oxidase (P450) resistance mechanisms were determined by pre-exposing *An. gambiae* and *An. funestus* to the oxidase inhibitor PBO at 50 μg/bottle for 1 h, followed immediately with exposure to discriminating doses of deltamethrin and permethrin-coated CDC bottles at 12.5 and 21.5 μg/bottle, respectively, for 30 min [25]. Resistance and intensity testing were not performed with carbamate insecticide, to which test mosquitoes were fully susceptible.

### Insecticide resistance intensity tests

Insecticide resistance intensity testing of *An. gambiae* and *An. funestus* to permethrin and deltamethrin was performed by exposing them to CDC bottles coated with serial dosages, first at the diagnostic dosages of 12.5 and 21.5 μg/bottle of deltamethrin and permethrin, respectively, and subsequently to doses of 2×, 5× and 10× the diagnostic dosages. The test bottles and insecticide concentrates were prepared and provided by CDC staff in Atlanta, Georgia, USA, developers of the CDC bottle bioassay method. One bottle was prepared for each concentration; approximately 25 mosquitoes were exposed to each diagnostic dose and each test included a control bottle of 10–15 mosquitoes.

### Interpretation of results of insecticide susceptibility tests

All mosquitoes that die within the diagnostic time period (30 min) when exposed to insecticide-coated bottles are susceptible to a tested insecticide. Test mosquitoes surviving beyond the diagnostic time threshold are assumed to have some degree of resistance. The most important information is the mortality at the diagnostic time, but the bioassay is monitored beyond the diagnostic time to evaluate the percentage of resistant mosquitoes. Interpretation of CDC bioassay results at 30 min is that < 95% mortality indicates resistance whereas WHO tube bioassays score suspected resistance at 90–97% and confirmed resistance at < 90% mortality [17, 20]. CDC bioassay tests are discarded if control mortality is > 10%. Abbott’s formula is used to correct results if the mortality at 2 h in the control bottle is between 3 and 10% [26].

### Determining insecticide resistance mechanisms

Increased production of detoxification enzymes in target insects can play an important role in insecticide resistance [17, 18]. Pre-exposing an insect to an enzyme inhibitor (synergist) will often overcome a targeted metabolic resistance mechanism and return such insects to near full susceptibility to a particular insecticide. Three outcomes are seen after testing: (a) return to full (or near full) susceptibility to the insecticide in the CDC bottle bioassay after pre-exposure to the synergist; (b) if resistance to the insecticide is only partially abolished then the metabolic mechanisms related to the synergist are only partially conferring the resistance and other mechanisms may also play a role; and (c) if resistance to the insecticide does not change with pre-exposure to the synergist (i.e., an increase in mortality is not observed), the metabolic mechanisms related to the synergist are likely not involved in the resistance observed [17].

### Determining insecticide resistance intensities

Resistance intensity testing is a relatively new procedure in use with the CDC bottle bioassay. Interpretation of tests demonstrating mosquito survival at 5× and 10× doses have yet to be correlated with control failure in association with LLIN or IRS use, but studies are underway to determine to what degree high intensity survival of field-collected mosquitoes is associated with operational failure.

## Results

**Fig. 2:**
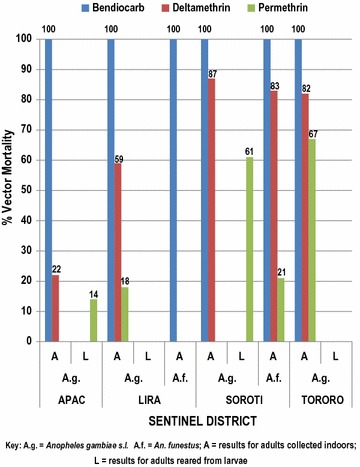
Percent mortality of *Anopheles gambiae* and *An. funestus* at 30-min diagnostic time after exposure to three insecticides in four sites in Uganda, June 2015

**Fig. 3:**
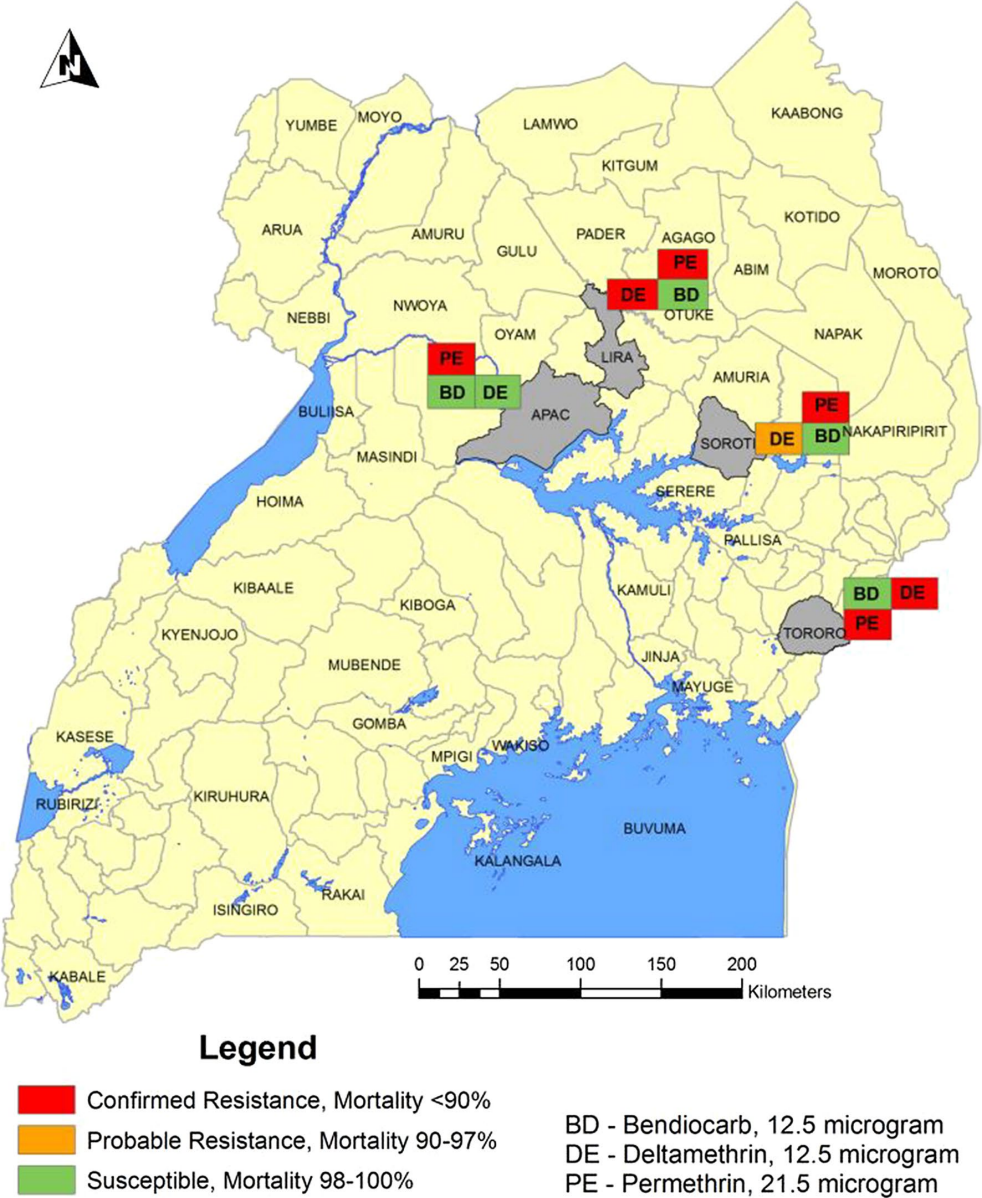
Map of Uganda showing insecticide susceptibility results using CDC bottle bioassay, June 2015

**Fig. 4:**
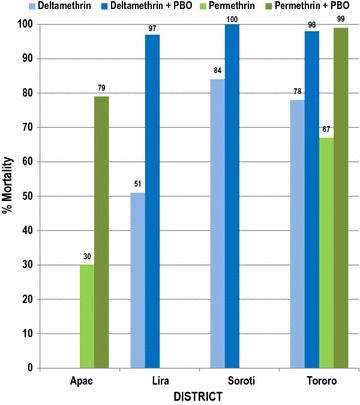
Percent mortality of *Anopheles gambiae* after 30-min exposure to diagnostic doses of permethrin alone and permethrin + PBO, deltamethrin alone, and deltamethrin + PBO in four districts in Uganda, June 2015

**Fig. 5:**
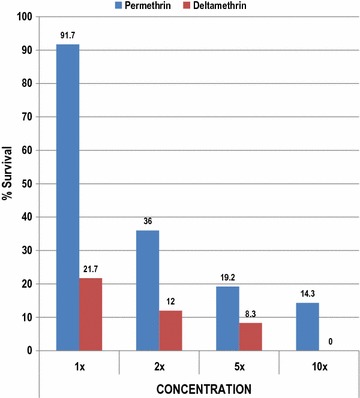
Percent survival of *Anopheles gambiae* exposed to permethrin and deltamethrin at different concentrations after the 30-min diagnostic time using the CDC bottle bioassay, Tororo District, June 2015

*Anopheles gambiae* was the only malaria vector tested in all the four study sites (Apac, Lira, Soroti and Tororo). *Anopheles funestus* was tested only in Lira and Soroti due to insufficient numbers to perform the test in the other two districts. The susceptibility/resistance levels in *An. gambiae* and *An. funestus* to the different insecticides, insecticide intensities and oxidative detoxification mechanism are shown in Tables 1, 2, 3, 4, 5 and Figs. 2, 3, 4, 5, while PCR speciation and *kdr* mutations of the *An. gambiae* complex are shown in Table 6. Table 3 summarizes mortality and resistance status of *Anopheles* from all study sites to diagnostic doses. The number of *An. gambiae* tested for each insecticide varied between 97 and 243, while the number of *An. funestus* tested for each insecticide varied between 58 and 207.

**Table 1:** Number and percentage of female *Anopheles gambiae* (*A.g.*) and *Anopheles funestus* (*A.f.*) killed after exposure to three different insecticides in Apac and Lira Districts, northern Uganda, June 2015

# dead at time (minutes)	Apac District				Lira District			
	Ben^a^	Del^b^	Per^c^	Del	Ben	Ben	Del	Per
	*A.g.*	*A.g.*	*A.g.*	*A.g.*	*A.g.*	*A.f.*	*A.g.*	*A.g.*
	A^d^	A	L^e^	A	A	A	A	A
0	0	0	0	0	0	0	0	0
15	102	11	5	1	102	134	0	0
30	102	32	14	8	102	134	54	24
Total tested	102	101	100	82	102	134	91	137
Percent mortality	100	31.8	14	9.7	100	100	59.3	17.5

^a^Bendiocarb

^b^Deltamethrin

^c^Permethrin

^d^A = live-captured adults

^e^L = field-collected larvae reared to the adult stage

**Table 2:** Number and percentage of female *Anopheles gambiae* (*A.g.*) and *Anopheles funestus* (*A.f.*) killed after exposure to three different insecticides in Soroti and Tororo Districts, eastern Uganda, June 2015

# dead at time (minutes)	Soroti District					Tororo			
	Ben^a^	Ben	Del^b^	Del	Per^c^	Per	Ben	Del	Per
	*A.g.*	*A.f.*	*A.f.*	*A.g.*	*A.g.*	*A.f.*	*A.g.*	*A.g.*	*A.g.*
	A^d^	A	A	A	L^e^	A	A	A	A
0	0	0	0	0	0	0	0	0	0
15	120	117	42	49	2	2	103	67	49
30	120	117	92	83	65	21	103	111	99
Total tested	120	117	111	95	106	101	103	136	147
Percent mortality	100	100	82.9	87	61.3	20.8	100	81.6	67.3

^a^Bendiocarb

^b^Deltamethrin

^c^Permethrin

^d^A = live-captured adults

^e^L = field-collected larvae reared to the adult stage

**Table 3:** Summary of percent mortality and resistance status of *Anopheles gambiae* (*A.g.*) and *Anopheles funestus* (*A.f.*) to three insecticides at four sites in Uganda, June 2015

Insecticide tested	Diagnostic dose	Apac		Lira			Soroti			Tororo	
	Source	*A.g.*		*A.g.*		*A.f.*	*A.g.*		*A.f.*	*A.g.*	
		A^a^	L^b^	A	L	A	A	L	A	A	L
Carbamate											
Bendiocarb	12.5 µg	100^c^		100^c^		100^c^	100^c^		100^c^	100^c^	
Pyrethroid											
Deltamethrin	12.5 µg	22^d^		59^d^			87^d^		83^d^	82^d^	
Permethrin	21.5 µg		14^d^	18^d^				61^d^	21^d^	67^d^	

^a^A = live-captured adults

^b^L = field-collected larvae reared to the adult stage

^c^Susceptible to insecticide

^d^Resistant to insecticide

**Table 4:** Number of adult female *Anopheles gambiae* (*A.g.*) killed after exposure to a diagnostic dose of permethrin and deltamethrin with and without PBO pre-exposure in four districts in Uganda, June 2015

# dead at time (minutes)	Apac		Lira				Soroti		Tororo			
	Per^a^ only	Per + PBO^c^	Del^b^ only	Del + PBO	Del only	Del + PBO	Del only	Del + PBO	Del only	Del + PBO	Per only	Per + PBO
	*A.g.*	*A.g.*	*A.g.*	*A.g.*	*A.g.*	*A.g.*	*A.g.*	*A.g.*	*A.g.*	*A.g.*	*A.g.*	*A.g.*
	L^d^	L	A^e^	A	A	A	L	L	A	A	A	A
0	0	0	0	0	0	0	0	0	0	0	0	0
15	0	14	7	24	10	26	20	20	23	34	49	59
30	19	48	63	30	39	61	69	29	66	43	99	86
Total tested	63	61	99	30	86	64	82	29	85	44	147	87
% mortality	30.2	78.7	63.6	100	45.3	95.3	84.1	100	77.6	97.7	67.3	98.9

^a^Permethrin

^b^Deltamethrin

^c^Piperonyl butoxide

^d^L = field-collected larvae reared to the adult stage

^e^A = live-captured adults

**Table 5:** Percentage survival of *Anopheles gambiae* exposed to permethrin and deltamethrin at different concentrations using the CDC bottle bioassay, Tororo District, June 2015

Insecticide	Concentration			
	1×	2×	5×	10×
Permethrin	91.7	36.0	19.2	14.3
Deltamethrin	21.7	12.0	8.3	0

**Table 6:** Distribution of *kdr*-East (L1014S) and *kdr*-West (L1014F) in *Anopheles gambiae* and *Anopheles arabiensis* mosquitoes from four sites in eastern Uganda

Site	*EAST*							*WEST*					
	Species	Genotype count				Allelic frequency		Genotype count				Allelic frequency	
		No.	RR	RS	SS	R	S	No.	RR	RS	SS	R	S
Apac	gam^a^	30	30	0	0	1.00	0.00	0	0	0	0	0	0
Lira	gam	62	61	1	0	0.99	0.01	0	0	0	0	0	0
Soroti	gam	8	8	0	0	1.00	0.00	0	0	0	0	0	0
Tororo	gam	16	16	0	0	1.00	0.00	0	0	0	0	0	0
Apac	arab^b^	43	0	0	43	0	1.00	1	1	0	1	1.00	0
Lira	arab	1	0	1	0	0	1.00	0	0	0	0	0	0
Soroti	arab	30	0	30	0	0	1.00	0	0	0	0	0	0
Tororo	arab	68	0	68	0	0	1.00	0	0	0	0	0	0

^a^gam = *Anopheles gambiae*

^b^arab = *Anopheles arabiensis*

Full susceptibility of *An. gambiae* to bendiocarb was observed in all the four study sites in Apac, Lira, Soroti and Tororo. Full susceptibility of *An. funestus* to bendiocarb was also observed in Soroti. Resistance (< 95% mortality) of *An. gambiae* to deltamethrin was observed in all the four study sites, with mortality varying from 22% in Apac to 87% in Soroti. Resistance of *An. gambiae* to permethrin was observed in all the four study sites, with mortality ranging from 14% in Apac to 67% in Tororo (Tables 1, 2, 3, 4, 5 and Figs. 2, 3, 4, 5). Susceptibility of *An. gambiae* to deltamethrin and permethrin differed significantly, but the mortality fell into the same susceptibility category (resistance, suspected resistance, susceptible) according to WHO criteria.

### Results of PBO-synergized bottles to suppress oxidase activity

Pre-exposure of *An. gambiae* to PBO for 1 h prior to exposure to discriminating doses of permethrin- and deltamethrin-coated CDC bottles restored the efficacy of these insecticides in Soroti and Tororo, indicating that oxidase enzymes were involved in the resistance of *An. gambiae* to both insecticides. However, pre-exposure of *An. gambiae* to PBO for 1 h prior to exposure to the discriminating doses of permethrin and deltamethrin-coated CDC bottles resulted in partial abolition of resistance to deltamethrin in Lira, and to permethrin in Apac, indicating that oxidase enzymes were partly responsible for insecticide resistance, while other mechanisms might also play a role (Table 4).

### Results of insecticide resistance intensity bioassays

Results for Tororo indicated that 2 out of 24, or 8.3%, of *An. gambiae* exposed to deltamethrin survived the 5× diagnostic dose, but that none survived the 10× diagnostic dose. Five out of 26, or 19.2%, and 3 out of 21, or 14.3%, of *An. gambiae* exposed to permethrin survived 5× and 10× diagnostic doses, respectively (Table 5).

### Occurrence and distribution of East (L1014S) and West (L1014F) knock-down resistance (*kdr*) point mutations in *Anopheles gambiae* in Uganda

Table 6 shows the result of PCR analysis for species in 258 *An. gambiae* mosquitoes collected from the four study sites. Of the 258 *Anopheles* examined, 142 (55%) were *Anopheles arabiensis* and 116 (45%) were *Anopheles gambiae* sensu stricto (s.s.). The 258 mosquitoes were further genotyped for *kdr*-east (L1014S) and *kdr*-west (L1014F) point mutations. Of the 116 *An. gambiae* s.s., 115 were homozygous for L1014S and one was heterozygous. Of the 142 *An. arabiensis*, 141 were homozygous for susceptible wild type and one was homozygous for L1014F. The distribution of L1014S and L1014F mutations in *An. gambiae* s.s. and *An. arabiensis* in the four study sites is shown in Table 6. L1014S was detected in *An. gambiae* s.s. in all study sites at allelic frequencies of 100, 99, 100 and 100% in Apac, Lira, Soroti and Tororo, respectively. No L1014S mutation was detected *in An. arabiensis*. L1014F point mutation was detected only in *An. arabiensis* in Apac. These results show that the L1014S is associated with *An. gambiae* s.s. only in the study sites while L1014F is associated with *An. arabiensis* in just one site.

## Discussion

Past observations with WHO tube tests in some of the current study sites indicated high resistance in malaria vectors to pyrethroids but susceptibility to carbamates and organophosphates [5, 6].

This study conducted in June 2015, gave similar results at the same sites to a study conducted in 2014 with respect to bendiocarb using the CDC bottle bioassay, consistently killing all exposed *An. gambiae* mosquitoes within 15 min post-exposure. *Anopheles gambiae* mortality to deltamethrin from Soroti in June 2015 was at 87% but only 22% in Apac. The Soroti site served as a control monitoring site, never having received IRS, but having received LLINs as have all other Uganda districts. In Tororo, permethrin killed 67% of *An. gambiae,* but only 14% in Apac (Table 3). Tororo District had only recently begun IRS in late 2014 and LLINs were widely distributed throughout the district. Rice farming occurs in Tororo and insecticides sold for agricultural pests are available in town (Tororo). Resistance in malaria vectors to permethrin and deltamethrin has remained high in all the study districts as shown by the present CDC bottle bioassay studies in June 2015. In other bottle bioassay studies in April and September 2014 and as with WHO tube bioassays in 2009, 2011 and 2013 [19], similar results were seen. Pyrethroid-resistant mosquitoes may negatively affect the performance of permethrin-treated LLINs used for malaria vector control. Moreover, the survival of exposed mosquitoes to 5× diagnostic doses of both deltamethrin and permethrin and 10× doses of permethrin might be an indicator of encroaching control failure of LLINs treated with these insecticides. This finding should urgently be confirmed through more-extensive studies using field-collected mosquitoes in cone bioassays on new nets treated with both insecticides.

A recent publication cited an increase in incidence of malaria in Tororo and the reduction of susceptibility to pyrethroids in local *Anopheles* populations as a possible factor for the waning effectiveness of LLINs in that area [27]. Although some variations in mortality were observed between tests performed on adults reared from larvae and those from the early morning adult mosquito collections, all mortalities fell within the same susceptibility category according to the WHO criteria [19, 20].

Lira District was a previous control monitoring surveillance site, however, it was included in the new group of IRS districts when IRS operations transitioned from northern Uganda to northern and eastern Uganda in late 2014 and early 2015. Lira was sprayed with bendiocarb during this time and subsequently rotated to pirimiphos-methyl in April 2016. Low mortality rates in *An. gambiae* to pyrethroid insecticides (59% to deltamethrin and 18% to permethrin) suggest agricultural pesticides and/or LLINs could be a driving force for selection of pyrethroid resistance in this mosquito. Two oxidase mechanism tests were run in Lira, one test raised mortality in *An. gambiae* exposed to deltamethrin from 63.6 to 100% while the second test raised mortality from 45.3 to 95.3%, demonstrating active oxidative detoxification mechanisms in this mosquito.

Apac District received IRS beginning in 2009 and continued through 2014, a total of six spray seasons. IRS transitioned from a pyrethroid insecticide in 2010 to a carbamate later that year and continued for the remaining five spray seasons. Apac District recorded the world’s highest entomological inoculation (EIR) rates of over 1500 infective bites/year during a 2002–2005 study [16]. LLINs are widely available throughout the district and subsistence agriculture is prevalent, although we do not know to what extent agricultural insecticides are used. This district consistently produces very low pyrethroid mortality rates (Table 3) and is now a control monitoring site. With the removal of IRS in 2014, we are monitoring this district to see if this removal will raise pyrethroid mortality rates or whether universal LLIN coverage will exert continued selection pressure on malaria mosquitoes and keep kill rates low. Oxidative mechanism testing raised mortality rates in *An. gambiae* to permethrin from 30.2 to 78.8%, showing the presence of oxidative and other resistance mechanisms from that district.

Results from insecticide resistance mechanism testing clearly indicate that oxidases played a role in the resistance of *An. gambiae* to pyrethroids, while other mechanisms might also play a role in the resistance of *An. gambiae* in Lira and Apac. Clearly, oxidative resistance mechanisms are prevalent in all four monitoring sites, illustrating possible beneficial effects from use of PBO-treated LLINs and organophosphate IRS insecticide application.

Intensity testing indicates that low-level (< 20% of test mosquitoes) high intensity resistance (5×, 10×) is occurring to LLIN pyrethroids at one location. MoH personnel have just recently begun intensity testing with plans to expand from the four IRS zone surveillance sites to eight national monitoring sites this year with the intent of monitoring yearly. It is not yet clear what 5× and 10× resistance means in terms of operational compromise, the test is new and more data is needed, especially cone testing of colonized 5× and 10× survivors. The level of resistance intensity (i.e., percent survival at twice, 5× and 10× the diagnostic dose of insecticide used to test for resistance as measured by the CDC bottle bioassay) may provide important information on when insecticide resistance may compromise the operational effectiveness of vector control interventions [20]. There is evidence from President’s Malaria Initiative (PMI) entomological work in Zambia that mosquito survival was witnessed in blood-fed mosquitoes exposed to 5× and 10× diagnostic doses in houses where LLINs were recently distributed [28]. These results indicate that it is not just the frequency of resistance in a mosquito population that is important but in fact the level of resistance intensity (e.g., seeing 7% of mosquitoes surviving at 5× the diagnostic dose) might be most important from an operational perspective [28]. Three pyrethroid insecticides (permethrin, deltamethrin and alphacypermethrin) are currently used as net impregnation insecticides. Exposed mosquitoes that survive 10× the diagnostic dose may indicate developing control failure of the insecticide to which the mosquitoes are exposed. Future resistance monitoring should emphasize intensity and mechanism testing now that pyrethroid resistance is well established and known to occur around the country. In areas where mosquitoes surviving a 10× dose of a pyrethroid are found, follow-on assays with WHO cones and fresh LLINs should be conducted to determine if this level of resistance intensity has an operational impact.

The study found a wide distribution of L1014S in *An. gambiae* s.s. and confirms the presence of L1014F point mutation in *An. arabiensis* in Uganda as also reported by Mawejje et al. [9]. The separate occurrence of mutations in the two malaria vectors may indicate differential exposure of the two vectors to the sources that create selection pressure to *kdr* resistance. Their difference in behaviour (with *An. gambiae* s.s. having a tendency for indoor resting and *An. arabie*nsis with tendency for outdoor resting) might explain (in part) observed differences in the occurrence of point mutations in the two malaria vectors in Uganda.

## Limitations

The time constraints of conducting this research during an active indoor residual spraying campaign limited the mosquito collecting activities to 3 weeks and during a time in which *An. funestus* occurred at low numbers in most collection sites.

## Conclusions

*Anopheles gambiae* is resistant to deltamethrin and permethrin in all four districts under study, while *An. funestus* is resistant to the same pyrethroids in Lira and Soroti, where it was tested, however, *An. gambiae* was fully susceptible to bendiocarb. These results partly guided the Ugandan Ministry of Health’s decision to import synergized LLINs as part of its procurement of Global Fund LLINs and also triggered the MoH to rotate IRS insecticides from lambdacyhalothrin (pyrethroid) to bendiocarb (carbamate) and more recently to pirimiphos-methyl (organophosphate). Intensity bioassay results may indicate encroaching control failure of permethrin-treated LLINs and may inform decision-makers on the choice of LLINs for the country, by deploying synergized LLINs where oxidative resistance is the major resistance mechanism in *An. gambiae* or with use of PBO synergized IRS insecticides, should any come to market soon. It should be noted that PMI does not recommend IRS spray with organophosphates in homes supplied with PBO LLINs. Increased oxidase activity within mosquitoes potentiates organophosphate effectiveness while PBO suppresses oxidase activity within mosquitoes.

Based on these results, there is an urgent need to conduct more-extensive, country-wide studies to document the extent of oxidase and other insecticide resistance mechanisms (esterase, glutathione *S*-transferase), as well as the intensity of resistance to the various insecticides used for IRS and in LLINs. The collected data will inform the development of insecticide resistance management strategies for Uganda [29–32]. This is particularly important in view of the universal LLIN coverage campaign in Uganda that is likely to accelerate the development and spread of pyrethroid resistance in malaria vectors. The WHO tube bioassay has historically been used to detect insecticide resistance at sentinel sites from around Uganda and use of this test for routine resistance monitoring should be continued. The CDC bottle bioassay is easy to use and useful in determining insecticide resistance mechanisms and resistance intensity levels from around the country. These tests have only recently been implemented and their use should continue.
